# Diazonium-functionalized thin films from the spontaneous reaction of *p*-phenylenebis(diazonium) salts†

**DOI:** 10.1039/c8ra00792f

**Published:** 2018-02-09

**Authors:** Nicholas Marshall, Andres Rodriguez, Scott Crittenden

**Affiliations:** Dept. of Chemistry and Physics, University of South Carolina Aiken 471 University Parkway Aiken SC 29801 USA nicholasm@usca.edu; Dept. of Physics and Astronomy, University of South Carolina 712 Main St. Columbia SC 29208 USA

## Abstract

Salts of the diazonium coupling agent *p*-phenylenebis(diazonium) form diazonium-terminated conjugated thin films on a variety of conductive and nonconductive surfaces by spontaneous reaction of the coupling agent with the surface. The resulting diazonium-bearing surface can be reacted with various organic and inorganic nucleophiles to form a functionalized surface. These surfaces have been characterized with voltammetry, XPS, infrared and Raman spectroscopy, and atomic force microscopy. Substrates that can be conveniently and quickly modified with this process include ordinary glass, gold, and an intact, fully assembled commercial screen-printed carbon electrode. The scope and convenience of this process make it promising for practical surface modification.

## Abstract 2

We present a surface functionalization procedure based on diazonium-functionalized thin films produced by spontaneous surface grafting of *p*-phenylene-bis(diazonium) cation.

## Graphical abstract

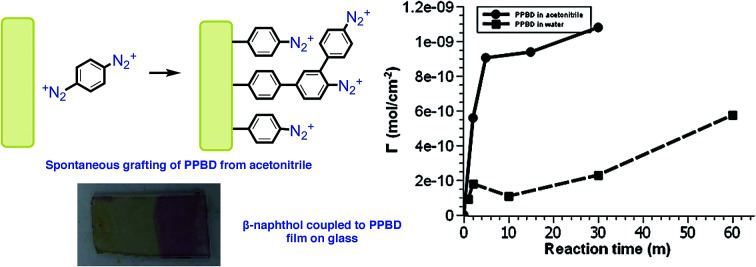

## Introduction

Surface modification is a ubiquitous problem in chemistry and materials science. In fields as broadly separated as photovoltaics^1^ and *in vitro* cell culture,^2^ it is necessary to treat a surface so that it presents a well-defined interface permanently covered in a particular functional group. The most well-known technique in this area is self-assembly of alkanethiol and alkylsiloxane/chlorosilane coating agents to prepare self-assembled monolayers (SAMs) These techniques are effective, but have limitations including instability at high temperatures in the case of thiols^3^ and long deposition times. Generally, to use self-assembly to make a functionalized surface, it is necessary to synthesize and isolate a thiol or silane with the desired functional group, requiring a substantial amount of work. Because of these and other limitations, other surface functionalization techniques have been developed. Among these, the runner-up to SAM formation may very well be electrografting using aryl diazonium salts.^4,5^ These highly reactive coupling agents are easily prepared from anilines and can be reduced at an electrode, giving a thin, durable polyarene layer which is attached to the electrode surface by some combination of covalent bonds and sheer insolubility in common solvents. Diazonium electrografting, therefore, clearly does not produce chemically well-defined layers as does self-assembly, but for most applied purposes the polymeric nature of the film is not a major drawback. Two major factors limit the applicability of aryl diazonium electrografting for surface modification; the need to use an applied reducing potential, and the highly acidic and oxidizing conditions used in preparing diazonium salts from anilines. Diazonium electrografting is thus generally limited to electrically conductive substrates and aromatic substrates with inert substituents. Although spontaneous grafting through thermal homolysis or photolysis of aryl diazonium salts over a long period of time is also known,^6,7^ the process is slower and some studies find that the films formed are not as durable as electrografted films. To immobilize complex or reactive species using diazonium electrografting, the need for extremely stable substituents on the aryl ring is generally worked around by deposition of the commercially available 4-nitrobenzenediazonium salt followed by reduction to give an aniline-functionalized thin film. The aniline group can then be modified by various well-known coupling chemistries such as diimide coupling or even a second diazotization step.^8^ Plenty of interesting surface architectures have been made in this way;^9–12^ however, it is desirable in preparative surface chemistry to minimize the number of separate steps needed to prepare a functional surface. Reactions which are quick in solution are much slower or even fail at an interface,^13^ and of course each step must be optimized separately. We therefore sought to design a simpler process of fewer steps for grafting-from reactions of diazonium salt layers.

**Scheme 1:**
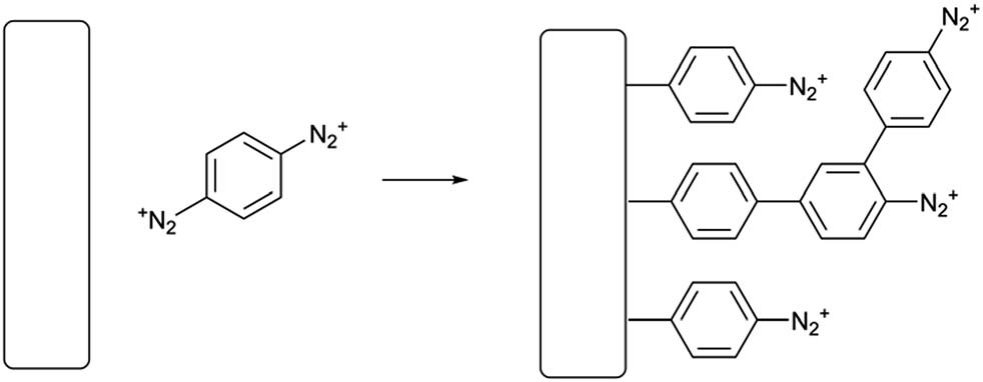
Spontaneous grafting of PPBD from an unreactive solvent such as acetonitrile.

**Scheme 2:**
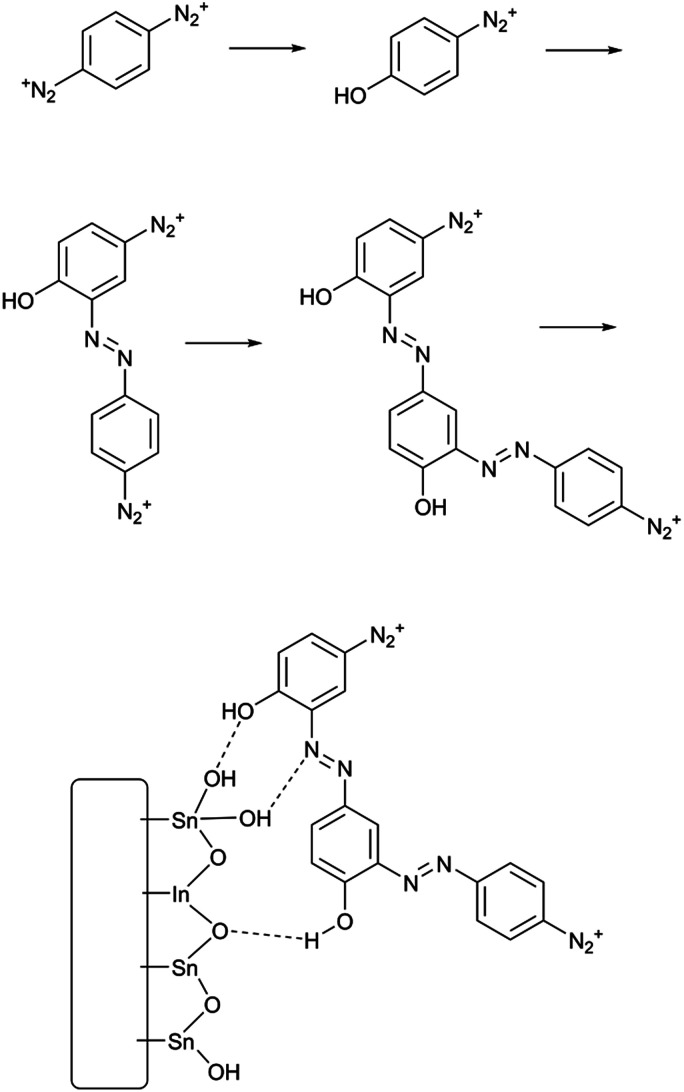
Solvolytic formation of phenolic azobenzene polymer from PPBD in water and its adhesion to an ITO surface.

In this work, we present a surface functionalization procedure of only two overall steps for several conductive and nonconductive materials based on the spontaneous surface grafting of a remarkable coupling agent, *p*-phenylene-bis(diazonium) cation (PPBD). We thoroughly characterize the thin films produced with this technique using voltammetry, UV-visible and infrared spectroscopy, X-ray photoelectron spectroscopy, and atomic force microscopy. Materials that can be modified include ordinary soda-lime glass, indium tin oxide (ITO), gold, glassy carbon, and a commercial screen-printed carbon paste electrode. Grafting of PPBD occurs through a solvent-dependent pathway, yielding a polyphenylene film in acetonitrile (Scheme 1) and a thick poly(azobenzene) film in water (Scheme 2). The solvolytic polymerization of PPBD with water is an unusual reaction which to the best of our knowledge has not been previously reported, although McCreery *et al.* have described the role of azo coupling in surface thin film growth^14^ while the DIAP procedure of Palacin *et al.*^15^ is similar in using a diazonium decomposition reaction to anchor a polymer film.

Our procedure is dramatically simpler than previously reported direct electrografting techniques for making diazonium-terminated films,^16,17^ and gives higher surface coverages of post-functionalization grafted groups. The procedure, to our knowledge, is the only single-step process reported to make diazonium-bearing thin films on both conducting and nonconducting substrates. Unlike diazo resins used for surface coatings,^18,19^ the grafting of PPBD produces a conjugated ultrathin film with control over the thickness of the final layer. Spontaneous surface grafting with PPBD produces a coating faster than most self-assembly methods, taking between 5–60 minutes to cover a surface. The technique can exceed a single monolayer in surface coverage and yields films with reasonably fast redox kinetics. While the synthesis of the coupling agent requires the use of acetic acid, the surface functionalization procedure itself can be carried out with no solvents other than water if the species to be immobilized is soluble in water, although the procedure works with nucleophiles in non-aqueous solvents as well. The procedure should complement existing techniques for producing diazonium-functionalized surface coatings, which generally require *in situ* diazotization of amine surfaces,^8^ electrografting,^16,17^ or synthesis of a complex linker.^10,20^ We believe the technique is immediately applicable to the construction of electrochemical and optical sensors, as well as other devices involving immobilized complex molecules.

## Experimental

### Materials

Indium tin oxide (ITO) coated slides and gold-coated slides were obtained from Sigma-Aldrich and NanoCS. *p*-Phenylenediamine was recrystallized from boiling water containing decolorized carbon and dried in a dessicator containing anhydrous calcium chloride pellets. 18 MΩ water from a central lab system was used in all applications requiring water. HPLC-grade acetonitrile and dichloromethane were stored over 4 A molecular sieves. Unless otherwise stated, chemical reagents were obtained from Fisher Scientific and used as received.

### Software

Except as noted below for IR baseline and export, software freely available to the general public was used as follows. General graphing was performed in SciDAVis 1.18.^21^ AFM image analysis was performed using the Gwyddion software package.^22^ XPS data was initially viewed and fits performed in XPSPEAK 4.1.^23^ Analysis of substrate surface area was performed by analysis of masked images in the FIJI distribution of ImageJ.^24^

### Instrumental methods

#### Electrochemical measurements

Cyclic voltammetry (CV) and alternating-current voltammetry was performed using a BASi PalmSens 3 potentiostat/galvanostat in a three-electrode cell configuration. Aqueous measurements were taken using 100 mM HCl or KCl as the electrolyte, and non-aqueous measurements were taken using a 100 mM solution of tetrabutylammonium hexafluorophosphate in acetonitrile. A platinum wire was used as a counter electrode. In all experiments save for those using pre-made screen printed electrodes, a silver wire, freshly washed in 12 M HCl and deionized water, was used as pseudoreference electrode. The ferrocyanide/ferricyanide redox couple appeared at 0.286 ± 0.003 V (measured repeatedly over continued use for one month) *versus* this pseudoreference in 100 mM HCl a bare gold working electrode. In the nonaqueous solution, the ferrocene/ferricenium redox couple appeared at 0.465 V *versus* this pseudoreference using a bare gold working electrode. The electrochemical cell was freed of oxygen by purging using a nitrogen bubbler before use. Electrochemical surface coverages were determined by integration of CV peaks; electrode surface areas were estimated by masking of the electrode using electroplating tape (3M, Inc.) and photographing the masked electrode with a 2.83 cm^2^ standard, followed by image analysis to determine the ratio of the mask area to the standard area.

#### Vibrational spectroscopy

Infrared spectra were collected using a Thermo Electron Nicolet 4700 Fourier transform IR spectrometer using a DTGS detector and fitted with a grazing angle accessory from the same manufacturer. Spectra were taken using *p*-polarized infrared radiation produced by fitting an inline KRS-5 linear polarizer. A grazing angle of 86° relative to the normal plane of the sample was used. A resolution of 4 cm^−1^ was used. Spectra were baseline corrected using manual baseline correction in the manufacturer's OMNIC software package.

#### X-ray photoelectron spectroscopy

XPS measurements were taken at the University of South Carolina's Center for Engineering and Computing, using a Kratos Axis Ultra DLD instrument equipped with a monochromated Al Kα source and hemispherical analyzer. On indium tin oxide substrates, the carbon 1s line was used as a binding energy reference and set to a value of 284.6 eV. On gold substrates, the gold Au 4f_7/2_ peak was used as a binding energy reference and set to a value of 84.0 eV. Fitting of peaks was performed with the XPSPEAK4.1 software package.

#### Atomic force microscopy

All AFM measurements were performed on a Dulcinea/Cervantes AFM from Nanotec Electronica using a silicon tip and cantilever operated in a noncontact “tapping” mode with an approximately 70 kHz resonance frequency.

#### Preparation of *p*-phenylenebis(diazonium) salts

Similarly to our previously published procedure,^16^ a solution of 1.4 g of sodium nitrite (20.3 mmol) was suspended in 15 mL (0.27 mol) of concentrated sulfuric acid, adding the nitrite in small portions at a rate slow enough to avoid the visible formation of brown nitrogen oxides. The solution was cooled in an ice bath. A solution of 1.0 g of *p*-phenylenediamine (9.2 mmol) in 10 mL of glacial acetic acid was added dropwise over 10 min, and the reaction was allowed to proceed for one hour. After the addition was complete, the reaction was stirred for 30 min. During this time, the reaction mixture turned into a thick slush. Note: this material is likely the bisulfate salt, which should not be isolated due to a potential explosion hazard. 21.4 mmol of either sodium tetrafluoroborate (2.35 g) or sodium hexafluorophosphate (3.60 g) was dissolved in a minimum amount of cold water, and this solution added dropwise with manual stirring using a glass rod to the reaction. 25 mL of ice-cold water was added. The thick off-white precipitate of bis(diazonium) salt was collected on a glass fritted funnel and washed with ice-cold water, cold methanol, and diethyl ether, and dried in a vacuum desiccator. When not in use, the salts were stored in a −20 °C freezer. However, both tetrafluoroborate and hexafluorophosphate salts could be stored on the bench in a sealed vial for days without any detectable degradation in the case of the tetrafluoroborate and with only minor cosmetic darkening that did not yield an impurity measurable by NMR in the case of the hexafluorophosphate. Yield (PF_6_-salt) 2.8 g (6.6 mmol, 72%). ^1^H NMR: 8.9 ppm (s) *vs.* TMS. UV-vis (water) *λ*_max_ 254 nm.

#### Surface modification with bis(diazonium) salts

A 50 mM solution of *p*-phenylenebis(diazonium) salt (PPBD-2PF_6_ or PPBD-2BF_4_) was prepared in acetonitrile, water, or the ionic liquid *bmim*-BF_4_. The solution or suspension was sonicated 30 seconds in a laboratory ultrasound bath and filtered through a 0.45 micron PTFE syringe filter. This filtration step should not be omitted, as suspended salt particles may adsorb to the surface and affect the following step.

ITO and gold substrates were cleaned by sonication in 95% ethanol for 5 minutes, blown dry in a stream of nitrogen, and treated with piranha solution in the case of gold (1 part by volume 30% aqueous hydrogen peroxide to which 3 parts by volume conc. sulfuric acid were added – CAUTION, potent oxidizer and burn hazard) for 5 minutes or by treatment in an argon plasma cleaner for ITO.

A freshly cleaned substrate (gold, indium tin oxide, or glass) was added to the diazonium solution in a clean 20 mL scintillation vial and allowed to stand for the indicated reaction time. The substrate was removed from the vial, rinsed with acetonitrile or water followed by acetonitrile, and transferred immediately to the second reaction solution. 0.1 M solutions of nucleophilic coupling partners were used in all cases (ferrocene in CH_2_Cl_2_, potassium iodide and hydroquinone in water, β-naphthol in water to which a minimum amount of saturated KOH solution had been added to induce dissolution). After 5 minutes, the sample was removed from the vial, rinsed with copious amounts of acetone, water, and ethanol, then sonicated for 10 minutes in dichloromethane. Due to the delicate nature of screen-printed electrodes, these were used as received without further cleaning and only aqueous solutions were used in deposition experiments, and solutions were drop-cast on the screen-printed working electrode and rinsed off with water only followed by blow-drying with a stream of nitrogen.

## Results and discussion

**Fig. 1:**
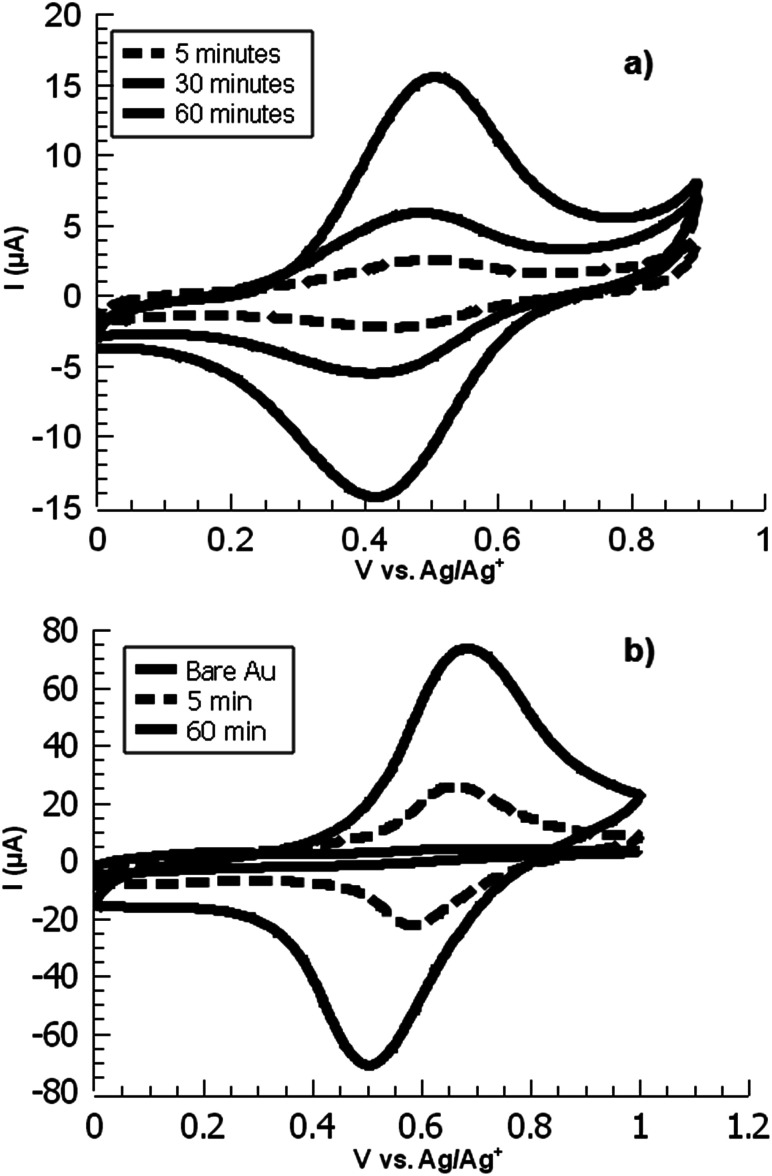
Overlaid CVs of ferrocene-functionalized PPBD films deposited from (a) water on ITO and (b) CH_3_CN on gold.

**Fig. 2:**
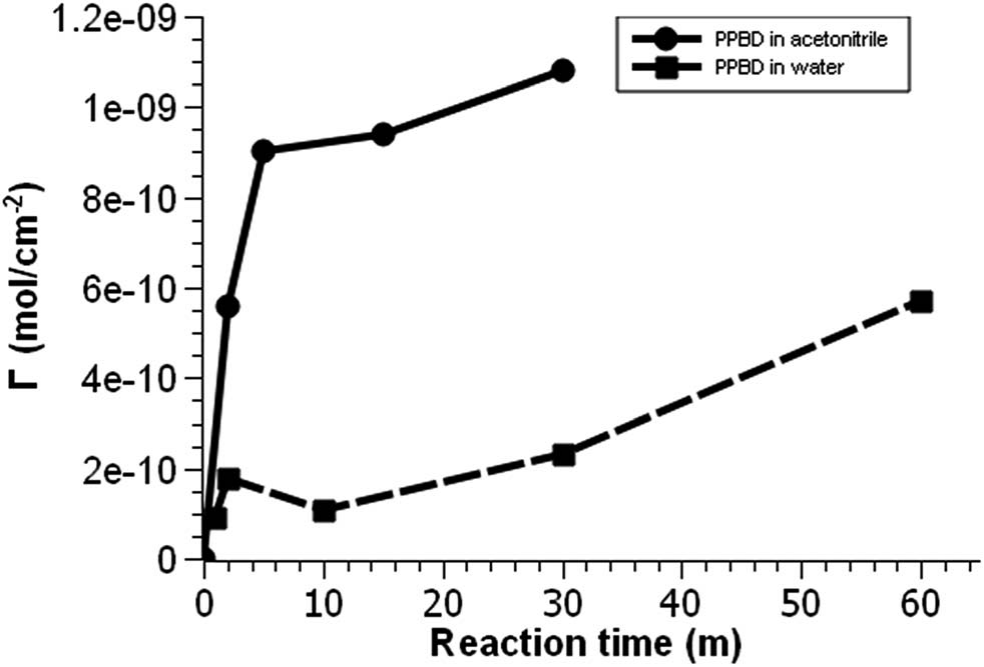
Voltammetric surface coverage of ferrocene-functionalized PPBD films as a function of time from CH_3_CN on gold (solid line) and from water on ITO (dashed line).

In electrochemical surface grafting of bisdiazonium salts, it has generally been found that the resulting thin films are polymeric and composed of a variety of chemical linkages including azo-coupled units and bonds formed by direct arylation of an aromatic ring by an aryl radical.^25–27^ In the present study, we see a distinctly different pattern of reactivity on oxide surfaces as opposed to glassy carbon and gold. The latter substrates, which have both been demonstrated to directly react with aryl diazonium groups,^28–30^ are rapidly coated by PPBD as measured by ferrocene grafting and voltammetry (Fig. 1 and 2) giving a diazonium-bearing thin film which is strongly bound to the substrate. This process likely takes place through radical arylation steps to yield a phenylene film^26^ bound through a direct Ar-surface bond^31,32^ as represented in Scheme 1.

**Fig. 3:**
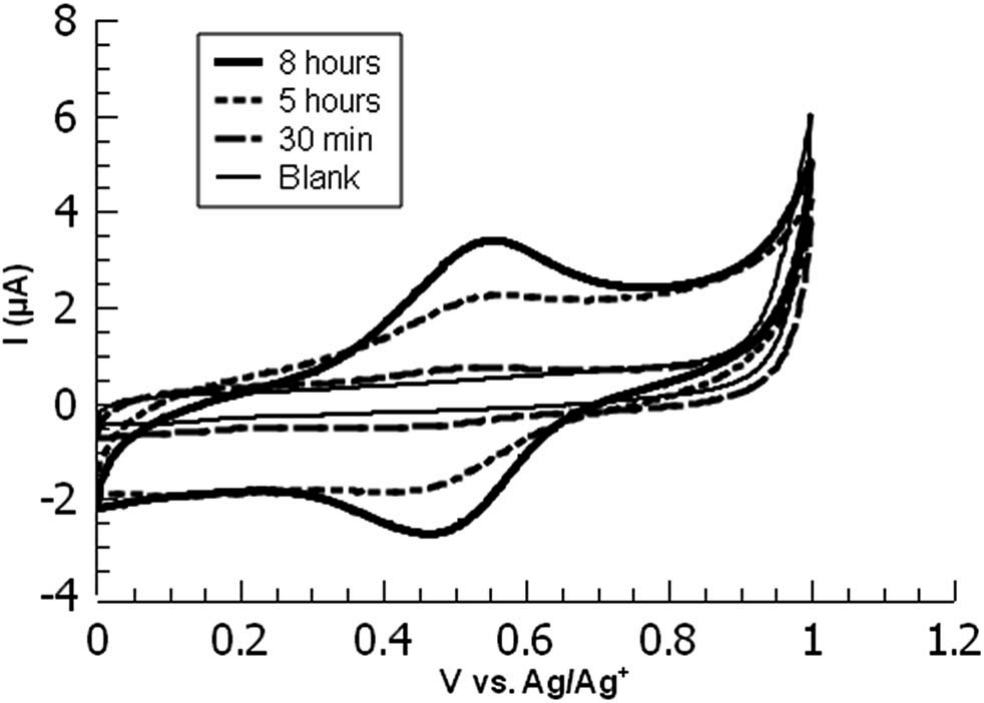
Overlaid cyclic voltammograms of ferrocene-functionalized PPBD films deposited from CH_3_CN on ITO at various timepoints.

**Fig. 4:**
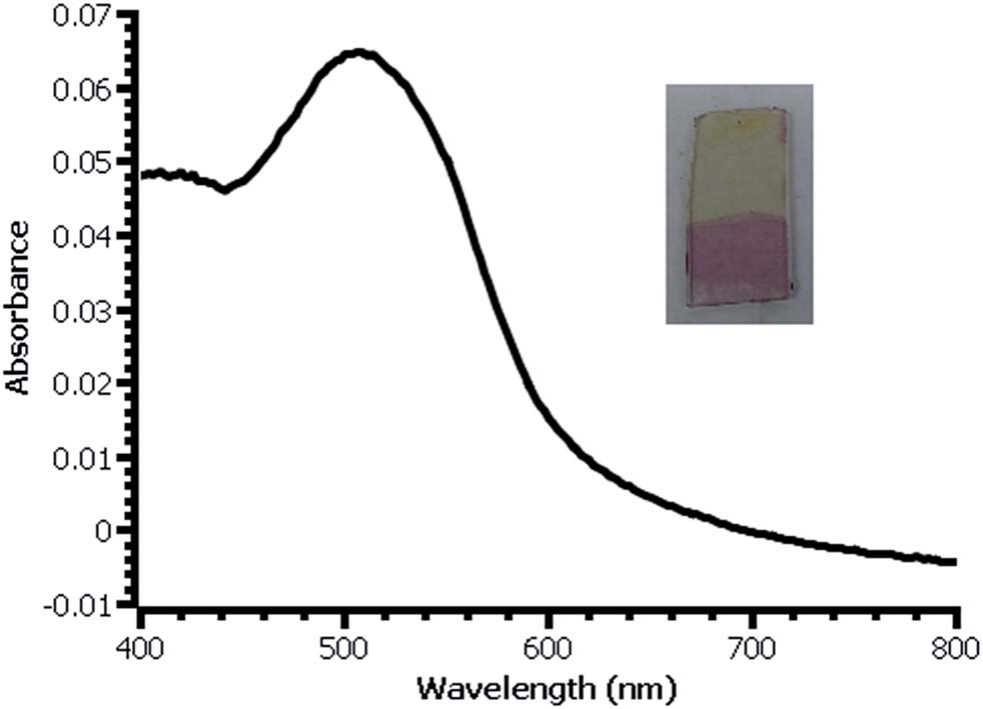
UV-vis spectrum of β-naphthol modified film deposited on glass slide from water. Inset: photograph of slide with modified (bottom) and unmodified (top) film.

On the other hand, oxides (both ITO and glass) react with PPBD solutions much more slowly in acetonitrile solution (Fig. 3). This reaction probably occurs analogously to the reaction of 4-nitrobenzenediazonium salt,^7,25,33^ giving surface-bound aryldiazonium groups connected possibly through aryl-O bonds^34^ but more likely by strong physisorption.^35,36^ To coat oxide surfaces more aggressively, a solution of PPBD in deionized water can be used (Fig. 1). Azo coupling between PPBD in solution and existing surface-bound aryl groups is much faster in protic solvents than in acetonitrile, accelerating the rate of PPBD bonding to the surface through N=N linkages. Additionally, in water PPBD can undergo solvolysis at an appreciable rate to form the *p*-hydroxybenzenediazonium cation, PHBD.^37^ At the concentration of PPBD used, PHBD can likely undergo azo coupling itself with PPBD similarly to the self-reaction of *p*-nitrobenzenediazonium^38^ giving an azobenzene with one phenol and two diazonium functional groups. As the solution ages, this polymer can also be incorporated into the growing surface-bound polymer film both by chemisorption and crosslinking through surface-bound diazonium groups. Chemisorption of polyphenols is known to occur on oxide surfaces^39,40^ and is reasonable to expect in this case. As the adsorbed film ages, it is likely that it will crosslink by decomposition of the bound diazonium groups, further bonding it in place (Scheme 2). We attribute the thick, strongly yellow-colored film (Fig. 4) formed by PPBD decomposition in water to this combination of physisorption driven by H-bonding and cross-linking of the film by the post-physisorption decomposition of diazonium groups.

While ITO and glass surfaces are coated more slowly in non-aqueous solvents than in water, these surfaces still react with the PPBD salt in non-aqueous solution. ITO surfaces reach limiting coverage values in 5–8 hours. The films resulting from this process are thinner based on AFM scratch testing than those produced using an aqueous solution of PPBD salt (Fig. 3), and have a maximum surface coverage based on immobilized ferrocene groups of *ca.* 1.5 × 10^−11^ mol cm^−2^, approximately a tenth of values seen in close-packed monolayers on ITO.

**Fig. 5:**
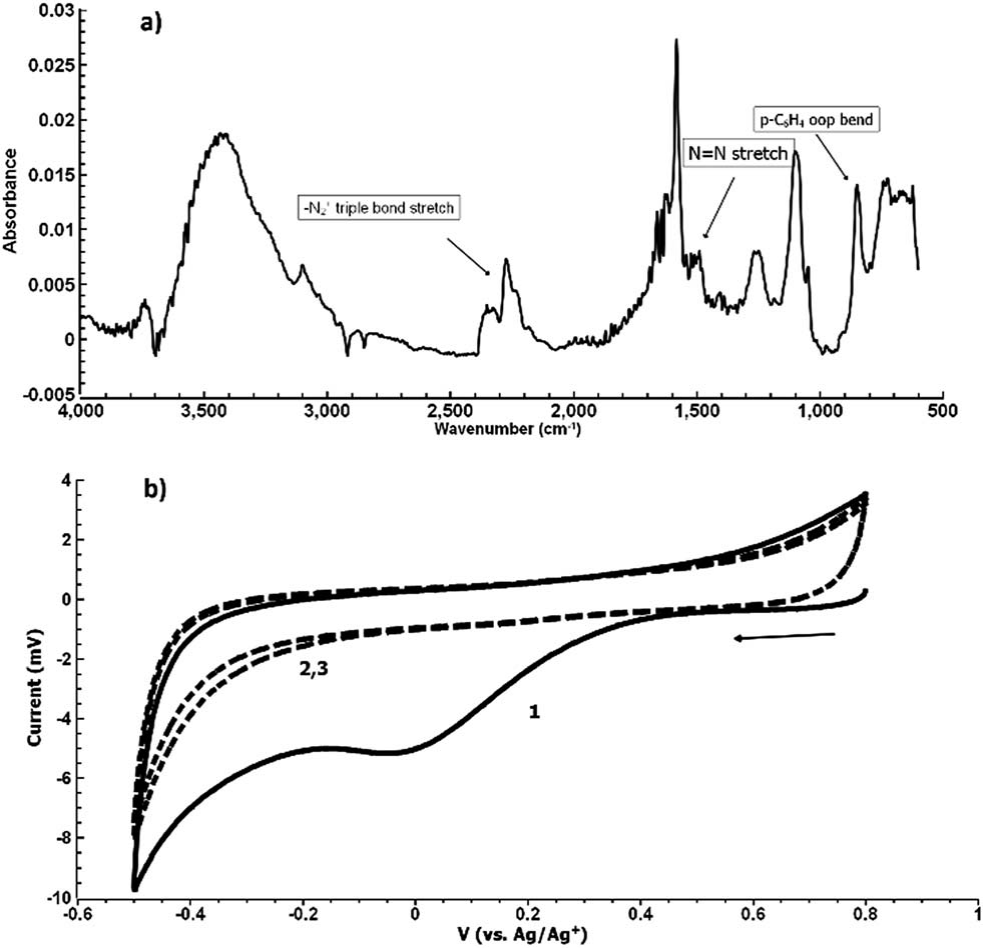
(a) Grazing-angle IR of a freshly prepared PPBD film deposited from water on Au. (b) CV of a freshly prepared PPBD film deposited from water on ITO in ACN/TBAPF_6_. The reduction of diazonium groups can be seen in the first wave near −0.2 V (solid line) and does not appear in subsequent sweeps (dashed lines).

**Fig. 6:**
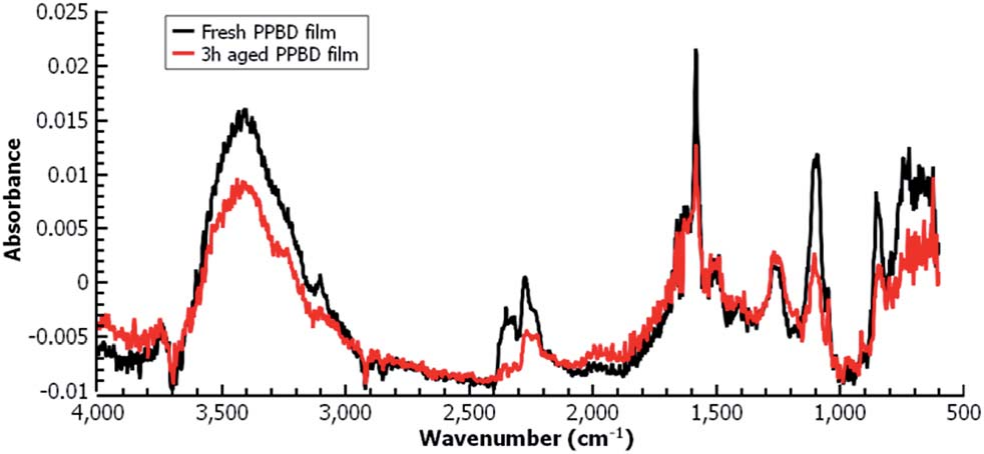
Grazing-angle IR of a fresh PPBD film deposited from water on Au (black) and the same film aged 3 h (red) in the laboratory environment.

Fig. 5(b) shows the electrochemical reduction of the diazonium group at the PPBD film surface. Reduction of the diazo group generates aryl radicals in the film, which irreversibly crosslink with each other. Because of the broad, irreversible peak shape, direct electrochemical surface coverage is hard to measure for this process but a direct estimate from integration of the peak gives 8.6 × 10^−11^ mol cm^−2^, reasonably consistent with the ferrocene surface coverage obtained from a film deposited under the same conditions (Fig. 2). The infrared spectrum of the PPBD film, Fig. 5(a), shows the distinctive stretching mode of a diazonium nitrogen–nitrogen triple bond stretch at 2250 cm^−1^. The proposed polyphenolic nature of the film deposited from water is consistent with the large O–H stretching peak near 3400 cm^−1^ and likely C–O stretch at 1090 cm^−1^. A weak peak near 1429 cm^−1^ is likely due to azobenzene linkages in the film, which are IR-forbidden in unsubstituted azobenzenes but can be seen in the IR spectrum when asymmetrically substituted. This absorption is blue-shifted relative to the IR-forbidden mode of azobenzene (1576 cm^−1^) due to the effect of the electron-donating hydroxy group. Fig. 6 shows IR of a fresh film and the same film aged 3 h in the laboratory environment. Roughly 50–80% of the intensity of the diazonium triple bond stretching peak has vanished after this time, but active diazonium groups are still present in the film. This result is reasonably consistent with the report of Downard *et al.*^43^ that approximately 50% of surface-bound diazonium groups on a 5 nm film produced by a two-step process on gold remain after 1 h. Both Downard *et al.* and Tour *et al.*^10^ have found that in highly conjugated molecules, spontaneous reduction of the diazonium groups by the substrate can occur instantly; this effect does not appear to be dominant on any of the substrates we examine in this work. On the other hand, irradiation of the PPBD film for 10 min with a common 254 nm UV lamp removes all traces of the N^2+^ triple bond stretching peak, consistent with known reactions of other diazonium-bearing polymers.^41^

**Fig. 7:**
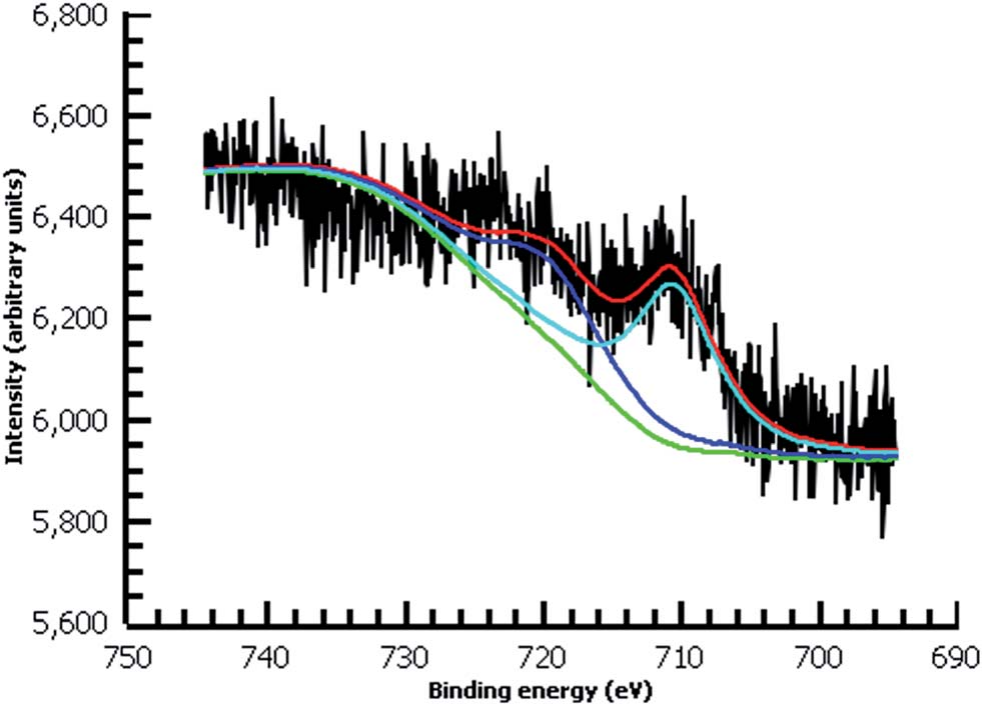
High-resolution Fe 2p XPS of diazonium film deposited 30 m from water on Au and reacted with 0.1 M ferrocene in dichloromethane.

**Fig. 8:**
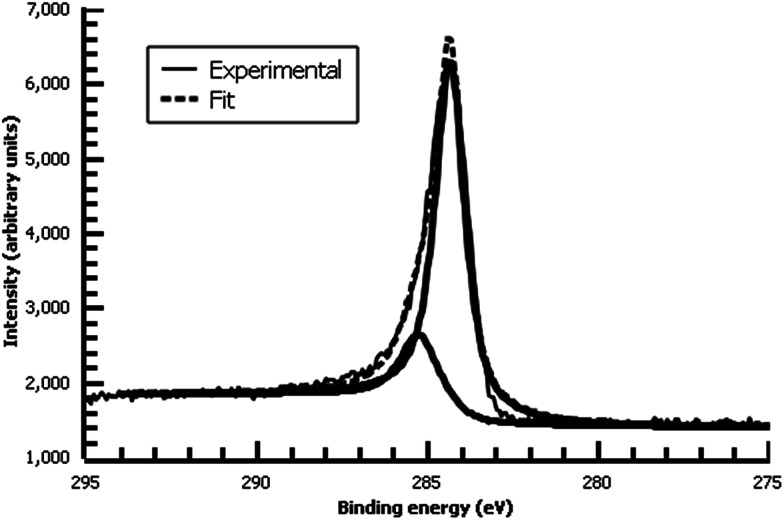
High-resolution C 1s XPS of diazonium film deposited 30 m from water on Au and reacted with 0.1 M ferrocene in dichloromethane.

**Fig. 9:**
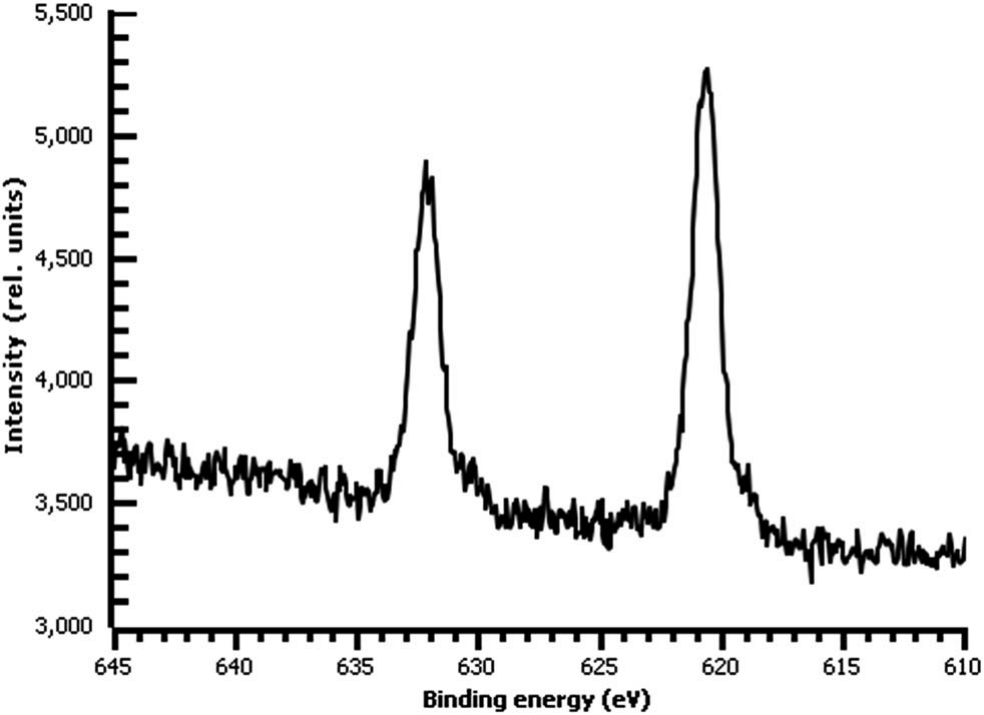
High-resolution I 3d XPS of diazonium film on Au deposited 30 m from water and reacted with 0.1 M aqueous KI.

A variety of nucleophiles can readily react with the PPBD film to become attached to the surface. Ferrocene reacts in an interesting Gomberg–Bachmann–Hey arylation with elimination of nitrogen to form a new C–C bond (Fig. 1). This reaction has been used in several reports to probe surface density of diazonium groups.^16,42,43^ Other aromatics with electron-donating substituents react in an azo coupling, giving azobenzene derivatives on the surface. Strong inorganic nucleophiles react in a free-radical process similar to the well-known Sandmeyer reaction to displace diazonium group. We reacted PPBD films with ferrocene, β-naphthol, hydroquinone, catechol, and potassium iodide to produce functionalized films in each case. In the case of ferrocene, the iron atom can be seen in XPS (Fig. 7), while the carbon spectrum shows C=C and C–O peaks but does not show any evidence of a low-binding-energy C–Au peak (Fig. 8). Iodine is incorporated in the film after reaction with KI (Fig. 9), and the dramatic two-electron proton-coupled redox reaction of immobilized hydroquinone can be observed by CV after coating of a commercial screen-printed conductive carbon ink electrode with PPBD in water, followed by reaction with aqueous hydroquinone (Fig. 12). It is not surprising that PPBD can modify a carbon-based material, as many examples of diazonium-based functionalization techniques exist for conductive carbons.^44–46^ However, it is particularly useful that PPBD in water can be used to modify these electrodes, which are quickly destroyed by contact with organic solvents. Similarly to applications of diazo resins, this PPBD-based coating technique could be used to immobilize an enzyme on a disposable electrode for a quick and easy route to an integrated electrochemical biosensor.

**Fig. 10:**
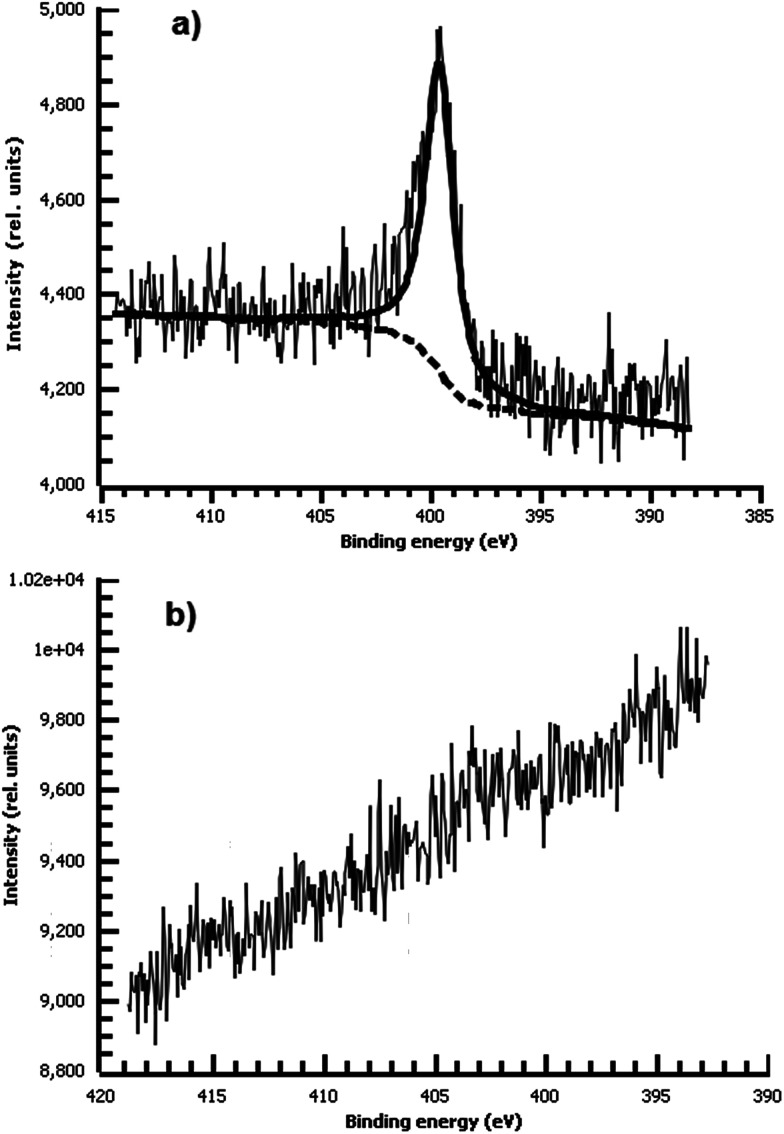
(a) High-resolution N 1s XPS of diazonium film deposited 30 m from water on Au and reacted with 0.1 M ferrocene in dichloromethane. (b) High-resolution N 1s XPS of diazonium film deposited 30 m from acetonitrile on Au and reacted with 0.1 M ferrocene in dichloromethane. Only a trace of nitrogen is detectable.

Consistent with our hypothesis that direct arylation is the dominant process from aprotic solvents while azo coupling dominates in water, the film deposited from water shows a strong N peak due to the N=N bond, while the film from acetonitrile shows no measurable nitrogen (Fig. 10). We did not observe any spontaneous film formation on ITO from the ionic liquid *bmim*-BF_4_.

**Fig. 11:**
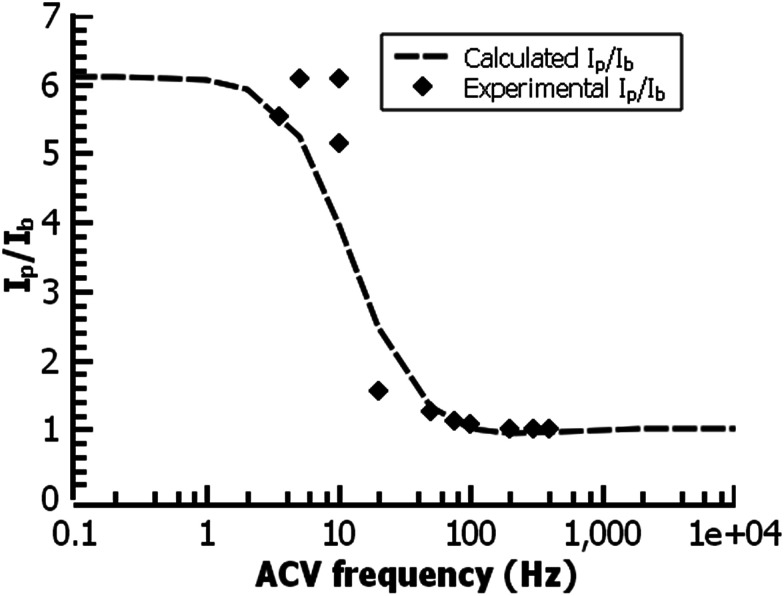
ACV analysis of a ferrocene-functionalized film deposited from ACN (30 min.) on ITO. The fit gives a rate constant for the redox reaction of 30 s^−1^.

In the film deposited from water on ITO, the ferrocene redox wave resembles that of other reported surface-bound ferrocene-containing polymer films, except that the film in the present study has little peak-to-peak separation and has a relatively fast electrochemical rate constant (*k* = 30 s^−1^) by ACV, faster than other ferrocene-containing polymers^47^ and comparable to ferrocene-bearing alkyl monolayers of only 1–2 nm in thickness.^48^ (Fig. 11) PPBD films deposited both from water and organic solvents should yield fully conjugated structures forming a relatively low barrier to charge carrier movement.

**Fig. 12:**
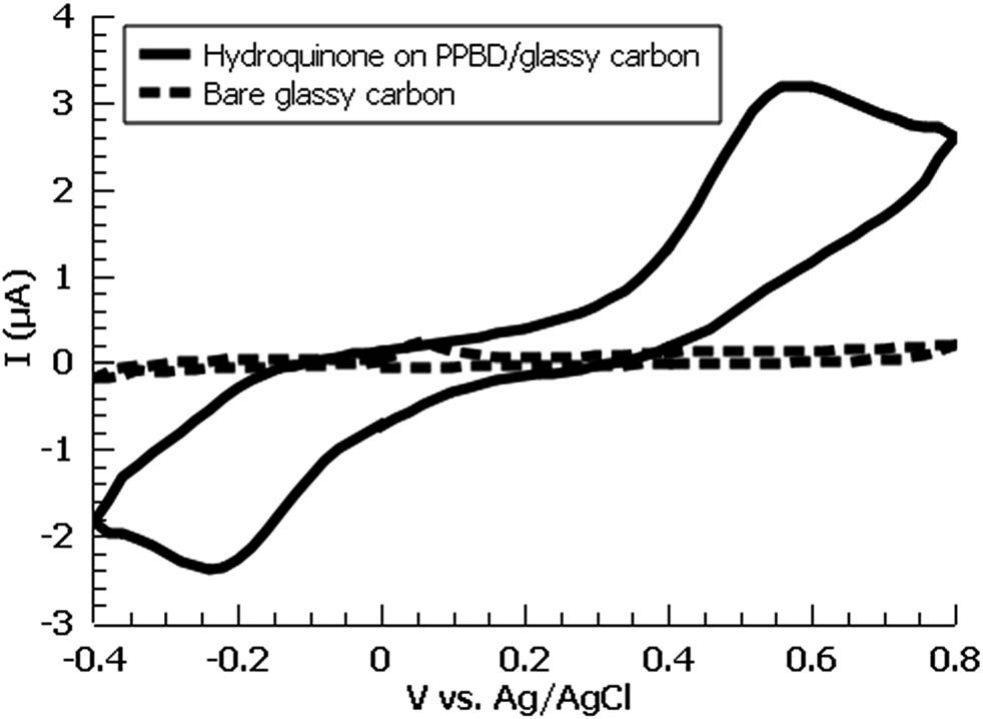
Cyclic voltammogram of hydroquinone-functionalized PPBD film deposited from water (5 min PPBD deposition time) on a commercial glassy carbon electrode at various timepoints. Blank electrode is also shown (dashed). Electrolyte is 0.1 M KCl in deionized water.

**Fig. 13:**
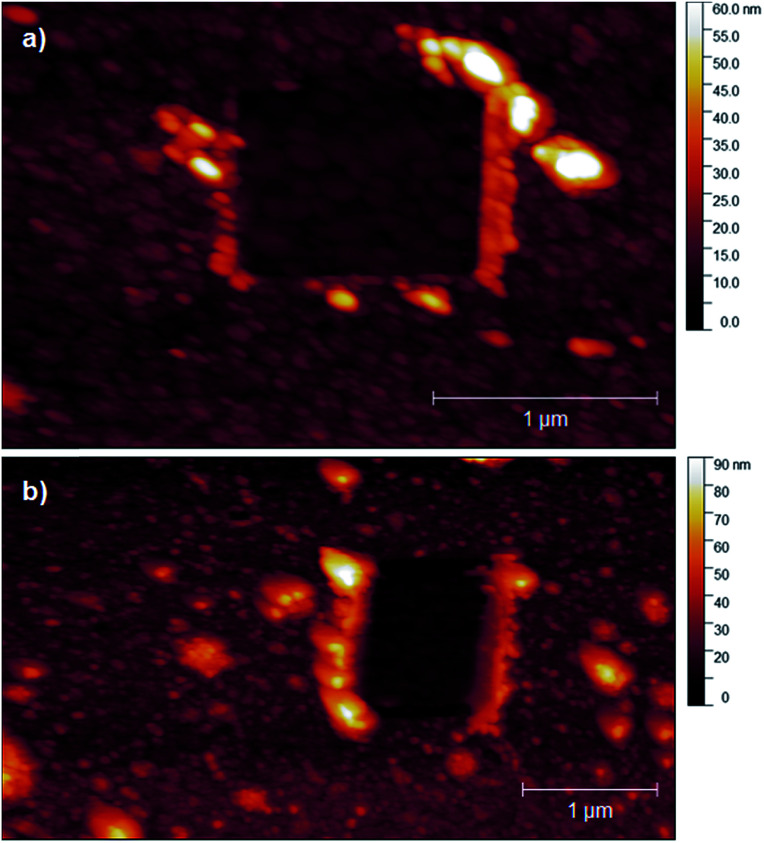
Atomic force microscopy of ferrocene-functionalized PPBD films on gold from CH_3_CN. Images include regions laid bare by scratching with the AFM tip. (a) 5 minutes of deposition time, film thickness based on scratch *ca.* 1 nm. (b) 60 minutes of deposition time, film thickness based on scratch 8 ± 2 nm.

PPBD deposition from water nears an early plateau for active functional groups detectable by CV in only 2 minutes, while the deposition from ACN on gold is nearly complete after 5 minutes (Fig. 3). In both cases, the surface coverage is similar to values for a monolayer on ITO or gold respectively.^48,49^ Interestingly, after a period of relative stability, coverage continues to increase beyond the highest value reported in literature for a close-packed monolayer on either ITO or gold. We attribute this super-monolayer surface concentration to the presence of a polymer thin film covered in ferrocene groups, with the 3-dimensional roughness of the film allowing a “skyscraper” packing of the groups on the surface. The rms roughness of the film increases from *ca.* 2 nm with 5 min PPBD deposition time (a roughness indistinguishable from the substrate, and consistent with the presence of a thin uniform film) to 6–8 nm with 60 min PPBD deposition time, yielding a film with an increased surface area (Fig. 13).

PPBD deposition on ITO surfaces from acetonitrile is much slower, as previously noted. This is a reasonable result in light of the greater stability of PPBD in acetonitrile, but yields the significant implication that ITO does not directly reduce PPBD under these reaction conditions as do metals or conducting carbon. This result is consistent with ITO's well-known use as a hole-injection material with organic compounds, and is reminiscent of other findings of complex interfacial interactions between ITO and organic thin films where ITO's conductivity is found to be due to surface states rather than electrons occupying a high-energy conduction band.^50^ Other reports of aryl diazonium grafting on ITO do not mention spontaneous reaction.^36^

In films deposited from acetonitrile, the nanoscale structure of the surface eventually becomes smoother than the underlying oxide or gold (Fig. 13 and S3†), consistent with the hypothesis that the surface produced by PPBD deposition is a uniform polyarene thin film similar to that seen in reductively-driven diazonium film deposition experiments.^51,52^ Interestingly, this film has a higher electrochemical surface coverage at long reaction times. Because of the rigidity of polymers containing phenylene^53,54^ or azobenzene^55,56^ backbone groups, it is unlikely that functional groups contained in the deep interior of the polymer would be accessible for reaction, so we believe that electrochemically measurable ferrocene groups are likely located near the surface of the polymer film. The higher surface coverage may be due to the increase in surface area due to the appearance of the finer-grained features (Fig. 13).

In thicker films, the color produced by reaction with β-naphthol is dramatic and visible (Fig. 4). Estimation of the surface coverage of coupled β-naphthol from the film's absorbance using an estimated molar absorptivity of 2.5 × 10^4^ gives a value on the order of 1 × 10^−9^ mol cm^−2^. Ferrocene coverage of a film prepared under similar conditions is 5.7 × 10^−10^ mol cm^−2^, reasonably close to the β-naphthol coverage; the difference between these values most likely falls within the uncertainty of the two measurement techniques. The difference in surface coverages may also be due to the difference in surface coupling efficiencies or variation between the films from experiment to experiment.

## Conclusions

Diazonium-functionalized thin films can be directly deposited in a single-step process on a wide variety of surfaces including glass, ITO, conductive carbon ink, and gold from solutions of the coupling agent *p*-phenylenebis(diazonium) (PPBD). These films can be reacted in a second step with various organic and inorganic nucleophiles to obtain functionalized surfaces. When acetonitrile is used as solvent, coating takes place through direct arylation of the surface by the diazonium salt, yielding a film composed primarily of polyphenylenes. Oxide surfaces do not participate in this reaction very rapidly, but can be quickly coated by solvolysis of PPBD with water as the solvent. In the latter case, the coating appears to be a poly(hydroxyazobenzene) film produced by azo coupling of phenolic groups formed by partial hydrolysis of PPBD in solution. Coatings 50–60 nm thick can be obtained with PPBD in water, while films from acetonitrile can reach 6–8 nm in thickness. Both techniques allow for surface coverage of functional groups greater than that of a single self-assembled monolayer. The rate constant of redox reactions occurring in immobilized species is reasonably rapid, on the order of 30 s^−1^. The reported surface modification techniques using PPBD solutions are easy and quick, and may find use in a variety of applications in sensor design and materials science.

## Conflicts of interest

The authors declare no conflicting interests.

## Supplementary Material

RA-008-C8RA00792F-s001
