# Supplementary material for: Diazonium-functionalized thin films from the spontaneous reaction of p-phenylenebis(diazonium) salts
Source: RSC Adv. 2018 Feb 9;8(12):6690–8. doi: 10.1039/c8ra00792f (PMC9078370; doi:10.1039/c8ra00792f)
Supplement: RA-008-C8RA00792F-s001 [file RA-008-C8RA00792F-s001.pdf]

Electronic Supplementary Material (ESI) for RSC Advances.
This journal is © The Royal Society of Chemistry 2018

Supporting information for

Diazonium-functionalized thin films from the spontaneous reaction of *p*-phenylenebis(diazonium) salts

Nicholas Marshall,[a] Andres Rodriguez,[a] and Scott Crittenden[b]

a. Dept. of Chemistry and Physics, University of South Carolina Aiken, 471 University Parkway, Aiken, SC, 29801, USA.
b. Dept. of Physics and Astronomy, University of South Carolina, 712 Main St. Columbia, SC, 29208, USA.

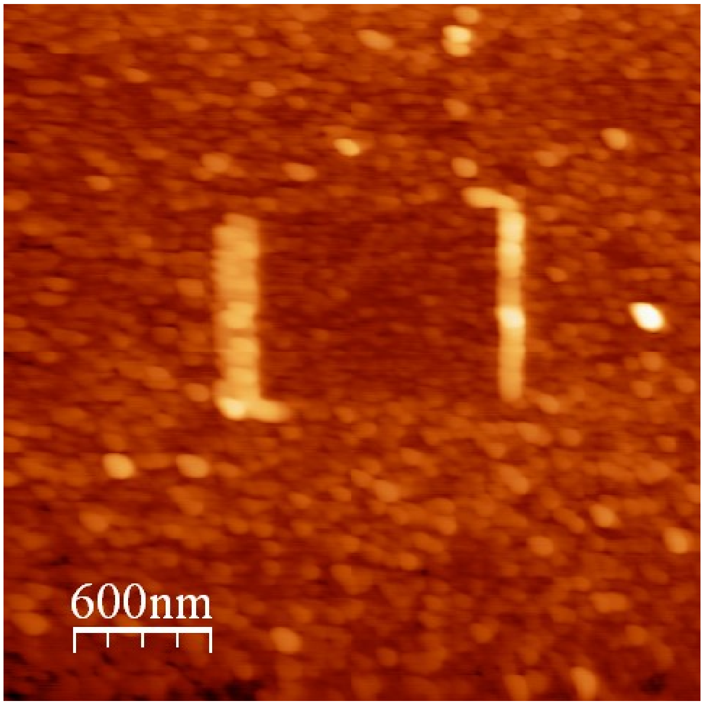

Figure S1. AFM (with scratched region) of a ferrocene-functionalized PPBD film deposited from ACN (5 min.) on ITO. Scratch depth 2 nm.

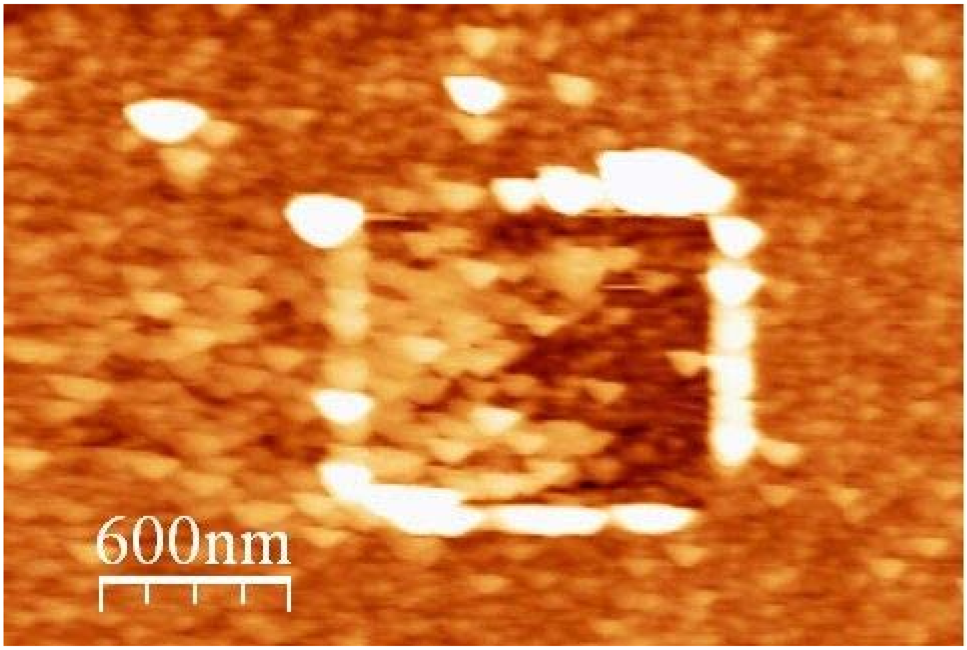

Figure S2. AFM (with scratched region) of a ferrocene-functionalized PPBD film deposited from water (5 min.) on ITO. Scratch depth 4 nm.

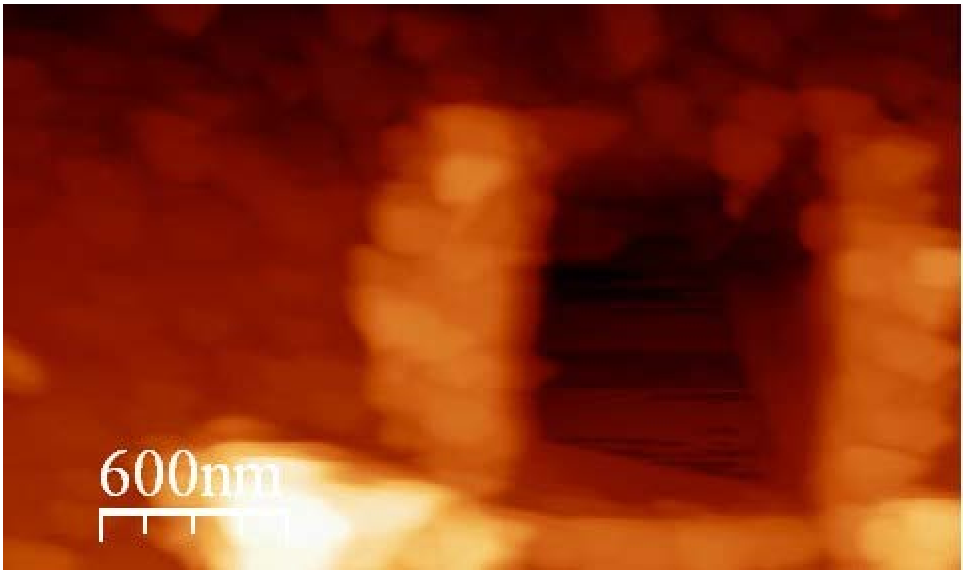

Figure S3. AFM (with scratched region) of a ferrocene-functionalized PPBD film deposited from water (60 min.) on ITO. Scratch depth 60 nm.

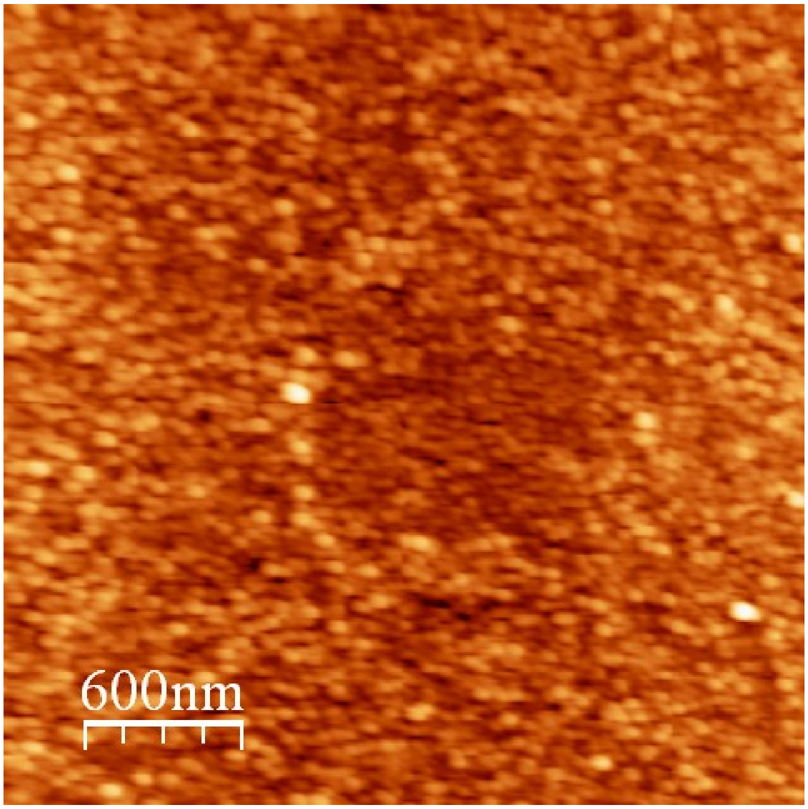

Figure S4. AFM (with scratched region) of cleaned, bare ITO, showing very little distinction between scratched and unscratched areas.

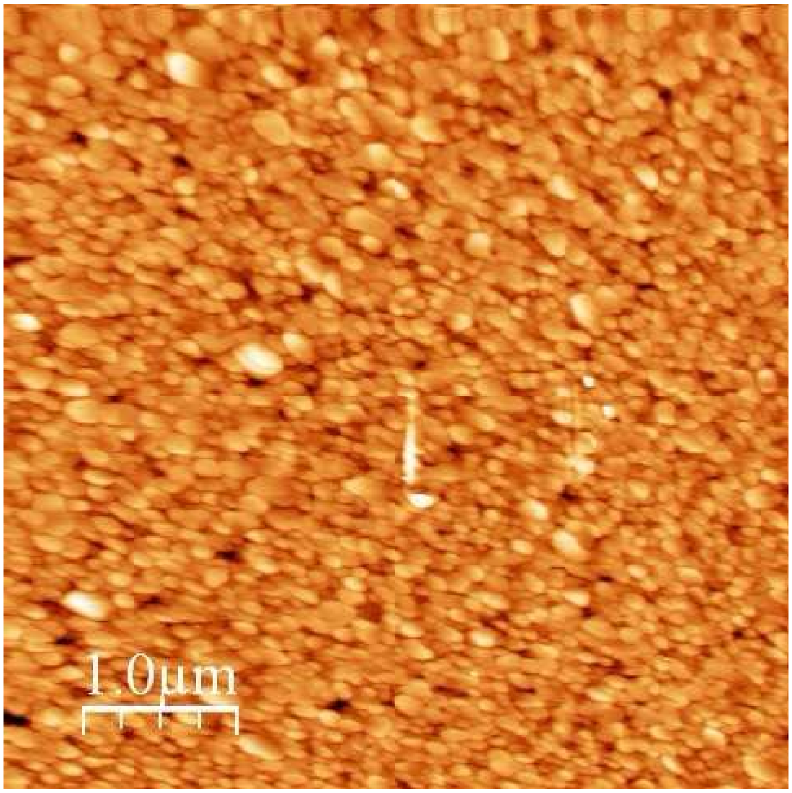

Figure S5. AFM (with scratched region) of cleaned, bare gold surface, showing very little distinction between scratched and unscratched areas.
